# Impact of COVID-19 on HIV service delivery in Miami-Dade County: a mixed methods study

**DOI:** 10.1186/s12913-022-08849-8

**Published:** 2022-12-03

**Authors:** Audrey Harkness, Vanessa Morales, Wayne Defreitas, Pranusha Atuluru, Jahn Jaramillo, Elliott R. Weinstein, Daniel J. Feaster, Steven Safren, Raymond Balise

**Affiliations:** 1 School of Nursing and Health Studies, University of Miami, 5030 Brunson Drive, Miami, FL 33146 USA; 2 Department of Public Health Sciences, University of Miami Miller School of Medicine, Miami, FL USA; 3 Department of Psychology, University of Miami, Coral Gables, FL USA; 4 Department of Medicine, University of Miami Miller School of Medicine, Miami, FL USA

**Keywords:** COVID-19, HIV prevention, HIV treatment, Implementation, Service delivery

## Abstract

**Background:**

Facilitating access to HIV prevention and treatment is imperative in Miami-Dade County (MDC), a U.S. HIV epicenter. With COVID-19, disruptions to these services have occurred, leading HIV organizations to innovate and demonstrate resilience. This study documented COVID-19 related disruptions and resilient innovations in HIV services within MDC.

**Methods:**

This mixed methods cross-sectional study included HIV test counselors in MDC. In the quantitative component (*N*=106), participants reported COVID-19 impacts on HIV service delivery. Data visualization examined patterns within organizations and throughout the study period. Generalized estimating equation modeling examined differences in service disruptions and innovations. In the qualitative component, participants (*N*=20) completed interviews regarding COVID-19 impacts on HIV services. Rapid qualitative analysis was employed to analyze interviews.

**Results:**

Quantitative data showed that innovations generally matched or outpaced disruptions, demonstrating resilience on HIV service delivery during COVID-19. HIV testing (36%, 95%CI[28%, 46%]) and STI testing (42%, 95%CI[33%, 52%]) were most likely to be disrupted. Sexual/reproductive health (45%, 95%CI[35%, 55%]), HIV testing (57%, 95%CI[47%,66%]), HIV case management (51%, 95%CI[41%, 60%]), PrEP initiation (47%, 95%CI[37%,57%]), and STI testing (47%, 95%CI[37%, 57%]) were most likely to be innovated. Qualitative analysis revealed three orthogonal themes related to 1) disruptions (with five sub-components), 2) resilient innovations (with four sub-components), and 3) emerging and ongoing health disparities.

**Conclusions:**

HIV organizations faced service disruptions during COVID-19 while also meaningfully innovating. Our findings point to potential changes in policy and practice that could be maintained beyond the immediate impacts of COVID-19 to enhance the resilience of HIV services. Aligning with the US *Ending the HIV Epidemic Plan* and the National Strategy for HIV/AIDS, capitalizing on the observed innovations would facilitate improved HIV-related health services for people living in MDC and beyond.

## Background

Miami-Dade County (MDC) has been a United States (US) epicenter of the COVID-19 pandemic [1]. Within MDC and beyond, Black and Latino/a/x populations have been disproportionately impacted by COVID-19 [2, 3] due to social determinants of health, such as employment in essential, public-facing occupations, differential loss of health insurance, and unequal testing distribution [4–6]. In MDC, Black and Latino/a/x individuals had higher COVID-19 hospitalization rates (23.8% and 64.2%) compared to non-Latino/a/x White individuals (8.6%). Despite representing only 16.6% of MDC’s total population [7], Black patients accounted for 26% of COVID-19 related deaths.

Simultaneous to the COVID-19 pandemic, MDC continues to be a domestic epicenter of another infectious disease, HIV [8]. MDC’s HIV epidemic is also driven by disparities. In 2018, new HIV diagnoses in MDC occurred disproportionately among men who have sex with men (MSM; 68%), Latino/a/x (59%), and Black (28%) residents [9]. Given these disparities, the *Ending the HIV Epidemic* (EHE) plan [10] articulated the urgent need to scale up and disseminate evidence-based tools for HIV prevention (i.e., pre-exposure prophylaxis/PrEP, HIV testing) and treatment (i.e., antiretroviral treatment/ART) to individuals most affected by HIV in MDC and other high priority regions.

However, efforts to scale up and disseminate these evidence-based HIV services may be disrupted by COVID-19. Social distancing and stay-at-home orders impeded provision of services as organizations limited hours and faced closures [11]. COVID-19 related disruptions to HIV services observed in the literature to date include reduced HIV treatment and prevention service availability [12], discomfort to patients who are accustomed to in-person services [13], and limited staff due to providers being temporarily reassigned to COVID-19 related roles [12].

Yet, clinics, providers, and the healthcare industry have rapidly evolved during COVID-19 to meet patients’ needs, demonstrating the resilience of healthcare systems. Rapid innovation and resilience in healthcare delivery is what is needed to achieve EHE goals [10] and overcome disparities. Although innovation can refer to developing interventions and health service delivery models that did not previously exist (e.g., development of technology for home-based HIV testing), innovation can also refer to new *implementation* of existing interventions and health service delivery models that were not previously employed or viewed as feasible (e.g., a specific organization innovating their previously on-site only services to include home-based HIV testing availability). Furthermore, we use the terms “resilient innovation” to refer to innovations that emerged as an adaptive response to stress put upon the healthcare system and providers (e.g., COVID-19). To date, resilient innovations to HIV services in response to COVID-19 include: implementing remote services such as telehealth, home testing kits, and mailed prevention materials (e.g., condoms, medications), minimizing in person interaction and potential treatment disruptions by increasing prescription refill quantities, relaxing follow-up procedures, and implementing flexible pharmacy delivery methods [2]. For example, an HIV clinic in Missouri switched to audio only landlines instead of videoconferencing for patients without access to smartphones and mailed HIV medications directly to patients [14]. Similarly, in MDC, remote service delivery options such as mailed PrEP and relaxed requirements for HIV medication refills facilitated safe and easier access to HIV services during COVID-19 [15, 16]. Other organizations increased curbside resource distribution and mailed self-test HIV kits to clients [11, 17].

Given the ongoing need for resilient innovation in response to COVID-19 and other emerging disruptions to healthcare delivery [18], the current study, which took place within the first two years of the COVID-19 pandemic (April 22, 2020-February 1, 2022), sought to document: (1) COVID-related disruptions in HIV services in MDC and (2) innovations that may facilitate the resilience of HIV service delivery systems during and beyond COVID-19. COVID-19 is not the last infectious disease pandemic or major disruption to HIV services that healthcare systems, providers, and patients will face, therefore, this study has implications for a range of future potential scenarios in which HIV service delivery must resiliently adapt to challenging circumstances.

## Methods

### Participants and procedures

Participants in the parent study (the Referral Through Testing study) included HIV test counselors in MDC. Eligible participants were HIV test counselors in MDC certified by the Florida Department of Health. Although all participants were HIV test counselors, it is possible that they also had other roles within their organization (e.g., PrEP navigator, HIV case manager); however, given the brevity of this survey, other roles were not assessed. The parent study identified barriers and facilitators to referring HIV testing clients for PrEP and behavioral health treatment in the context of an HIV testing session. We added questions about how COVID-19 affected service delivery, which are the focus of the quantitative portion of this analysis (*N*=106 survey responses with COVID-19 questions). The parent study was a cross sectional study with data used in the current analyses collected from April 2020 to February 2022.

Additionally, as part of the parent study we recruited a subset of participants (*N*=20) to complete qualitative interviews. The parent study sought to examine referral determinants; the focus of the current secondary analysis is on the added interview questions exploring the impact of COVID-19 on HIV services. Interviews were collected from March 2021 to October 2021.

Participants were initially recruited through in-person appeals at test counselor re-certification trainings. However, with COVID-19, in-person trainings were discontinued, and we transitioned to recruitment efforts including flyers, email, social media, and snowball recruitment. There were approximately 600 certified HIV test counselors in MDC at the time of our study; assuming that all counselors were reached by our recruitment efforts, we had a response rate of approximately 22%. We recruited a subset of survey respondents for interviews. Our interview recruitment efforts were guided by the goal of achieving representation from as many different organizations as possible. Compensation was $10 for surveys and $20 for interviews, except for counselors from one organization with a policy against research incentives.

### Measures

#### Demographics

Survey participants completed demographic questions including years as an HIV test counselor, organization, and number of HIV tests administered in the past three months. Demographic questions were limited due to the brief nature of the survey. Interview participants completed additional demographic questions about themselves, and the individuals served by their organization.

#### Quantitative measures

Participants completed a checklist of disruptions and innovations to HIV-related services due to COVID-19. The checklist was adapted from a measure developed by the International Association of Providers of HIV Care [19]. Participants reported whether their organization experienced COVID-19 related disruptions to any of the following services: HIV testing, STI testing, PEP initiation, PrEP initiation, ART initiation, viral load testing, lab monitoring for STIs, HIV case management, sexual/reproductive health, mental/psychosocial health, or substance use treatment. Although HIV test counselors do not necessarily provide some of these services directly (unless they have multiple roles within their organization), they are typically a central referral hub to other services within their organization and therefore, are aware of the extent to which these other services would have been disrupted or innovated. For instance, in MDC, counselors are trained and expected to make medical, prevention, and other supportive service referrals and linkages including STI testing, PEP initiation, PrEP initiation, and treatment initiation for those newly diagnosed with HIV [20]; similar training is in place in other regions [21, 22]. The central role of HIV test counselors to other services is further underscored by guidelines from the US Preventive Services Task Force, which state the importance of testing as a context for triage to additional HIV services, including both prevention and treatment [23]. As such, we elected to administer and analyze the full instrument. Counselors were then asked whether their organization developed new or innovative ways of delivering those services during COVID-19. Response options included yes, no, decline to answer (which HIV test counselors who did not know if a service had been innovated or disrupted could select), and not applicable (which could be selected if the organization did not offer the service).

#### Qualitative interview

In semi-structured interviews, participants were asked how COVID-19 affected their organization’s operations, including disruptions and innovations to services. They were also asked specifically how COVID-19 affected PrEP and behavioral health treatment referrals during HIV testing sessions, given the parent study’s focus on referrals to those two services. Finally, interviewees were asked how COVID-19 affected the populations they serve. The interview questions were written by the first author and were directly informed by the research question for the current study (i.e., what were the COVID-related disruptions in HIV services in MDC and what were the COVID-related innovations that may facilitate the resilience of HIV service delivery systems during and beyond COVID-19).

### Research team

Informed by guidelines for enhancing the trustworthiness of our findings [24], we share key information about our research team involved in the analysis. Our team included students and faculty in public health, psychology, medicine, and data science/biostatistics. Team members varied in race/ethnicity and experience with HIV research and service delivery. Qualitative team members received interviewing and rapid qualitative analysis training, supplemented with team meetings to address questions and feedback.

### Data collection

Surveys were completed via REDCap. After reviewing and consenting in REDCap, participants proceeded to the survey. Procedures were approved by the University of Miami’s IRB. Interviews (~60 minutes long) were conducted remotely. Participants responded to another consent and demographic survey within REDCap prior to completing interviews.

### Data analysis

#### Quantitative analyses and visualizations

Data were examined using exploratory data analysis. Prior to analysis, we processed the data so only valid surveys relating to COVID-19 disruptions and innovations remained. This involved sub-setting the dataset, recoding variables, and excluding incomplete/spam responses. We received a total of 229 survey responses. Responses that did not include the COVID-19 questions relevant to this secondary analysis were removed from the data, leaving a sample of 112. Of the remaining survey responses, partially completed responses (*n*=1), and spam or duplicate responses (*n*=15) were also excluded, resulting in a total of 106 survey responses included for the secondary analysis.

There were two levels of nesting in the resulting data set. First, the data set had multiple questions about the occurrence of both disruptions and innovations around different services delivered; these repeated disruption/innovation questions are nested within each respondent. In addition, each respondent was nested within an organization and there were multiple respondents for most organizations. In addition, whereas this is a multi-level, cross-sectional survey, the surveys were completed at different calendar times and therefore at different phases of the COVID-19 pandemic experience. Due to the multiple levels of nesting noted above, we aggregated data to the different levels to provide descriptive detail and visualizations.

Participants’ responses were first aggregated within organizations. We computed percentages at the organizational level based on the number of services participants reported as disrupted or innovated. We also created indicators for whether any respondent within an organization endorsed each response variable (i.e., indicated “yes” to a particular service being disrupted or innovated). This allowed us to capture the proportion of respondents endorsing disruptions and innovations and the proportion of organizations responding to COVID-19 items and the nature of that response. We visualized these values in bar charts. This first set of analyses and visualizations examined responses across the whole study period and did not account for any potential differences in response across time.

Since our first set of analyses and visualizations did not parse out responses across time, our next step was to assess whether participants’ views of their organizations’ disruptions/innovations differed as a function of when individual test counselors completed their survey. Within five time periods (April-July 2020, January-February 2021, March-April 2021, June-September 2021, and October 2021-February 2022), we calculated the percentage of respondents endorsing a particular view about COVID-19 disruptions/innovations. For example, if there were 15 respondents (regardless of organizational affiliation) from April through July 2020, we calculated the percentage of those who endorsed each disruption and innovation. This allowed us to view inflection points and innovation trends relative to perceived disruptions.

To evaluate whether disruption and innovation responses differed by service type, we examined a parametric analysis using the unaggregated nested data. Initial examination of a mixed model including both a random effect associated with organization and a random effect for the respondent (to account for repeated questions for type of disruption or innovation) showed important variability for the respondent in both reported disruptions and innovations. The random effect for organization was extremely close to zero for disruptions and while slightly larger for innovation, the point estimate was considerably smaller than the associated standard error. Therefore, our final parametric analyses omitted the nesting factor associated with organization. Within this analysis we examined the relationship between COVID-19 disruption and innovation items at the population-averaged (marginal) level via generalized estimating equations (GEE) modeling for a binary response using a binomial distribution and logit link. We specified an exchangeable working correlation structure to account for correlations of repeated questions within participants and report confidence limits using the empirical standard-errors which are robust to misspecification of the correlation structure. We analyzed disruption and innovation items separately. We controlled for time (month at which the person responded). We selected the service with the lowest frequency of “yes” responses as the reference category (i.e., ART initiation for disruptions, lab monitoring for innovations). Analyses took place in R (4.1.1), RStudio (2021.09.0+351), and SAS 9.4. We used the *lme4* package [25] in R as well as the PROC GENMOD and PROC GLIMMIX procedures in SAS.

#### Qualitative analysis

Complementing our quantitative analyses, we conducted a rapid qualitative analysis [26, 27] using Hamilton’s guidelines to ensure efficiency and rigor. The first author developed an interview summary template and guidelines. To ensure accurate summaries, interviewers completed summaries, which were audited by a second team member, and finalized by the first author. The team used a matrix to facilitate efficient and systematic identification of themes. The matrix allowed analysts to view and analyze each domain (i.e., disruptions, resilient innovations, disparities) simultaneously across participants. Team members reviewed the matrix to independently identify themes. The first set of themes was based on 10 participants. After each participant batch was analyzed, the team reviewed and determined if new themes emerged. After 15 interviews were completed, we reached saturation, meaning no new themes emerged [28].

## Results

### Demographics

Complete demographics are in Table 1. Survey participants had worked as an HIV test counselor for an average of 5.93 years. They worked at 27 different organizations, including CBOs, hospitals, and practices that offer HIV testing in MDC. Of note, we assessed organizational affiliation; given the brevity of this survey, we did not further assess facility-specific information for participants (e.g., if a CBO had multiple facilities). Interview participants ranged from 22 to 65 years old. Most identified as male (65%) and a racial/ethnic minority (95%).

**Table 1:** Demographics for HIV Test Counselors in Miami Dade County, Florida

Variable	Test Counselors	
	Survey (*N* =106)	Interview (*N*=20)
Number of Organizations Represented (*n*)	27	15
Years Testing (*M*, *SD*)	5.93 (6.73)	4.90 (5.35)
Number of Tests in Past 3 months (*M*, *SD*)	72.72 (137.42)	67.40 (87.09)
Age (*M*, *SD*)	N/A	39.45 (12.82)
Race^a^	N/A	
African American or Black		10 (50.0%)
Asian or Asian American		1 (10.0%)
White (including White Hispanic/Latino)		9 (45.0%)
Native Hawaiian or other Pacific Islander		0 (0.0%)
Different racial identity		1 (5.0%)
Decline to answer		1 (5.0%)
Ethnicity^a^	N/A	
Hispanic or Latino		11 (55.0%)
Haitian/Creole or Afro Caribbean Black		4 (20.0%)
Not Hispanic, Latino, or Haitian/Creole or Afro-Caribbean Black		5 (25.0%)
Decline to answer		1 (5.0%)
Current Gender Identity^b^	N/A	
Male		13 (65.0%)
Female		5 (30.0%)
Trans		1 (5.0%)
Cisgender female		1 (5.0%)
Education Level	N/A	
GED (high school equivalent)		1 (5.0%)
Some College/University		9 (45.0%)
College/University degree		10 (50.0%)
Populations served^b^	N/A	
Youth (<18 years)		2 (10%)
Adults (≥18 years)		11 (55.0%)
Uninsured		2 (10.0%)
Non-English speaking (e.g., Spanish)		4 (20.0%)
English speaking		4 (20.0%)
Lower income		9 (45.0%)
Middle income		2 (10.0%)
Immigrants		2 (10.0%)
Cisgender individuals		1 (5.0%)
Transgender individuals		4 (20.0%)
Men		5 (25.0%)
Women		6 (30.0%)
Black/African American individuals		11 (55.0%)
Latino/a/x/Hispanic individuals		8 (40.0%)
Caribbean individuals including Haitian		3 (15.0%)
Heterosexual individuals		3 (15.0%)
MSM and sexual minority individuals		9 (45.0%)
Individuals experiencing homelessness		1 (5.0%)
People who use substances		1 (5.0%)
Individuals at greater likelihood of HIV acquisition		1 (5.0%)

^a^Participants were able to select multiple races or ethnicities. ^b^ Note that for these items, participants were able to write in their own response. For “current gender identity,” categories reflect participants’ self-identification. For “populations served” we created a checklist based on participants’ responses. Those who reported serving any of the populations listed are noted here

### Quantitative findings

**Fig. 1:**
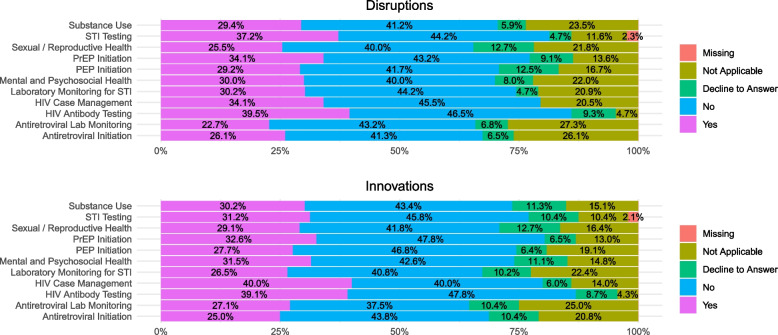
Organizations Reporting COVID-19 Disruptions and Innovations

First, we examined the overall distribution (across the whole study period) of service disruptions and innovations endorsed by participants within each organization. As shown in Fig. 1, the most disrupted services were HIV antibody testing (39.5% of organizations), STI testing (37.2%) PrEP initiation (34.1%), and HIV case management (34.1%). Participants most frequently reported innovations in their delivery of HIV case management (40.0%), HIV antibody testing (39.1%), PrEP initiation (32.6%), mental/psychosocial health services (31.5%), and STI testing (31.2%) in the context of COVID-19 (Fig. 1).

**Fig. 2:**
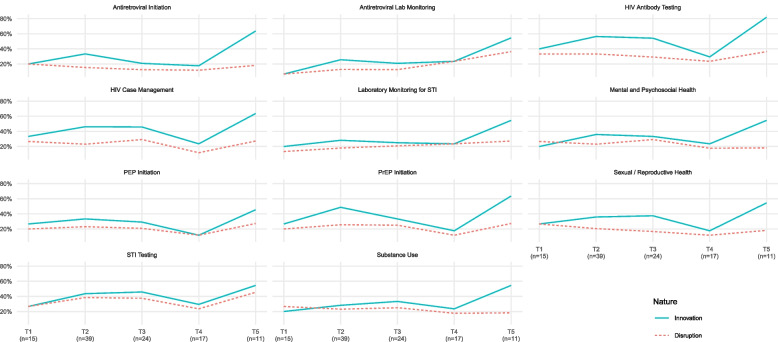
Comparison of Respondent Level Innovations to Disruptions

To understand patterns over time, we created Table 2 which shows the proportion of respondents at discrete time points within the study who indicated that each service type had been disrupted or innovated. Table 2 illustrates that both disruptions and innovations differed across the study period. To explore the relationship between time of data collection and disruptions/innovations, we visualized our data, creating line graphs comparing the overall amount of disruption and innovation with respect to each service over time (Fig. 2). Figure 2 shows that innovations generally matched or outpaced disruptions, suggesting a resilient response to COVID-19, whereby as disruptions occurred, organizations generally found ways to innovate to still be able to deliver needed services.

**Table 2:** Perceived COVID-19 Disruptions and Innovations Throughout Study Period

**Service**	**Apr-Jul 2020 (*N*=15)**		**Jan-Feb 2021 (*N*=39)**		**Mar-Apr 2021 (*N*=24)**		**Jun-Sep 2021 (*N*=17)**		**Oct '21-Feb 2022 (*N*=11)**	
	**Disruption**	**Innovation**	**Disruption**	**Innovation**	**Disruption**	**Innovation**	**Disruption**	**Innovation**	**Disruption**	**Innovation**
Antiretroviral Initiation	20%	20%	15%	33%	12%	21%	12%	18%	18%	64%
Antiretroviral Lab Monitoring	7%	7%	13%	26%	12%	21%	24%	24%	36%	55%
HIV Antibody Testing	33%	40%	33%	56%	29%	54%	24%	29%	36%	82%
HIV Case Management	27%	33%	23%	46%	29%	46%	12%	24%	27%	64%
Laboratory Monitoring for STI	13%	20%	18%	28%	21%	25%	24%	24%	27%	55%
Mental and Psychosocial Health	27%	20%	23%	36%	29%	33%	18%	24%	18%	55%
PEP Initiation	20%	27%	23%	33%	21%	29%	12%	12%	27%	45%
PrEP Initiation	20%	27%	26%	49%	25%	33%	12%	18%	27%	64%
Sexual / Reproductive Health	27%	27%	21%	36%	17%	38%	12%	18%	18%	55%
STI Testing	27%	27%	38%	44%	38%	46%	24%	29%	45%	55%
Substance Use	27%	20%	23%	28%	25%	33%	18%	24%	18%	55%

Table 3 displays the results of our two GEE models and displays parameter estimates, odds ratios, and probabilities for each service that showed a significant difference in likelihood of disruption or innovation compared to the reference service (i.e., antiretroviral initiation for disruptions and laboratory monitoring for innovations) based on the GEE models. After adjusting for the correlated outcome data, the GEE disruption model identified HIV testing (*p*=0.0002) and STI testing (*p*<.0001) as significantly more likely to be disrupted than the reference service (i.e., ART initiation, which was least disrupted). The disruption model also showed that averaged over the population, HIV testing was 1.89 (OR 95% CI: 1.35, 2.67) and STI testing 2.41 (OR 95% CI: 1.65, 3.54) time more likely to be disrupted than ART initiation during COVID-19.

**Table 3:** Parameter Estimates, Odds Ratios, and Probabilities for GEE Models

	**Est.**	**Lower**	**Upper**	***P*-value**	**OR**^a^	**Lower**	**Upper**	**π**	**Lower**	**Upper**
**Disruption**										
HIV Antibody Testing	**0.637**	0.302	0.972	*p*=0.0002	**1.89**	1.35	2.67	**0.372**	0.282	0.471
STI Testing	**0.881**	0.498	1.264	*p*<.0001	**2.41**	1.65	3.54	**0.429**	0.333	0.530
**Innovation**										
Sexual/Reprod. Health	**0.396**	0.017	0.759	*p*=0.04	**1.47**	1.02	2.14	**0.451**	0.352	0.555
HIV Antibody Testing	**0.866**	0.451	1.282	*p*<.0001	**2.38**	1.57	3.60	**0.570**	0.470	0.665
HIV Case Management	**0.606**	0.272	0.940	*p*=0.0004	**1.83**	1.31	2.56	**0.506**	0.407	0.604
PrEP Initiation	**0.462**	0.074	0.850	*p*=0.0196	**1.59**	1.08	2.34	**0.470**	0.372	0.569
STI Testing	**0.454**	0.075	0.833	*p*=0.0188	**1.58**	1.08	2.30	**0.468**	0.372	0.566

^a^The Odds Ratios compare the statistically significant variables against the disruption and innovation variable with the lowest frequency count (reference), which for the disruption dataset is Antiretroviral Initiation and for the innovation dataset is Laboratory Monitoring for Antiretroviral therapy

The GEE model exploring services most likely to be innovated (adjusting for correlated outcome data) identified five services that were significantly more likely to be innovated than the reference service (i.e., laboratory monitoring, which was least endorsed for innovation). Sexual and reproductive health services (*p*=0.04), HIV testing (*p*<.0001), HIV case management (*p*=0.0004), PrEP initiation (*p*=0.0196), and STI testing (*p*=0.0188) were all significantly more likely to be innovated during COVID-19 than the reference service. Significant odds ratios for innovations ranged from a low of 1.47 the odds (OR 95% CI: 1.02, 2.14) for sexual/reproductive health services to a high of 2.38 the odds (OR 95% CI: 1.57, 3.60) for HIV testing.

### Qualitative findings

Qualitative analyses identified three themes related to disruptions (with 5 sub-components), resilient innovations (with four sub-components), and ongoing and emerging health disparities. Themes are summarized with illustrative quotations (Q1, Q2, etc.) in Table 4.

**Table 4:** Qualitative Themes and Illustrative Quotations

**Themes**	**Quotations**
1. Disruptions due to COVID-19	
1.a. Reduction in staff availability and morale	Q1: “We were open. We never closed. We were hit very hard. We had almost 70% of our staff tested positive…Our regular time, the phone doesn’t stop, doesn’t stop. Imagine when we had 70% workin’ out. Imagine. We had used every single person for everything. The doctor was the medical assistant, was a doctor, was the phlebotomist. Imagine, he did everything.” (Test Counselor 1)
1.b. Disruptions to organizational networks	Q2: “We were not able to always get in contact with certain organizations because of COVID, that's like at the first couple of months, the way people were doing things changed significantly.” (Test Counselor 2)
1.c. Challenges transitioning to telehealth	Q3: “As far as everything else, it’s all been virtual, and that’s been really hard. It’s really hard to gauge anything with a client virtually. That’s the biggest barrier.” (Test Counselor 3)
1.d. Reduction in outreach and available services	Q4: “We're not able to provide the testings that we used to do. We used to go to different locations to provide testing, but because of the pandemic, we're really limited to what we're able to do.” (Test Counselor 4)
1.e. Reduction in client access and demand for services	Q5: “What has impacted us is people—we used to get more people to come in to be tested, but because of the fear of people being infected or more susceptible to be impacted by COVID, some people are very limited, and they don't—we don't have that many people coming to our center, only via appointments. It's getting a little better, but at the beginning…it was really difficult.” (Test Counselor 4)
2. Resilient innovations in the context of COVID-19	
2.a. Increased availability of telehealth to address healthcare needs during and beyond COVID-19	Q6: We're going to try, yes there's definitely patients who do, who did enjoy the lab box or did enjoy like the telehealth services and getting access to their [online medical] chart and things like that, so we have tried to keep that implementation, and then we also still offer it as an option so we're definitely trying to kind of broaden our horizons, in that way. (Test Counselor 5)
2.b. Increased availability of other remote service options	Q7: A client goes online, whether it be through an ad or a website and basically there’s a section there where they go click to sign up. They fill out some information first name, last name, phone number, email address a few other demographics there. They submit and then within 24 to 40 hours someone calls them by phone to do the pre-test questionnaire, a demographic questionnaire, and the risk factor questionnaires that would typically be done in the office. Then they get an at home HIV testing self-kit. If they live nearby the office they can actually pass by and drop it off. Or they can also test in their car if they don’t mind driving to the location, that’s option one. Option two which is the most popular is that the counselor themselves actually goes to the residence and drops it off at their home. Obviously, you know no contact, so they stay in their car or they just drop it off the porch or something like that they do that. Or the third option, which it really depends on distance, would be mailing it to them using the…HIV self-testing website as a way to have it delivered.” (Test Counselor 6)
2.c. Flexible methods of community-based outreach and engagement	Q8: “We go into the chat room [using social media or dating apps like Grindr], not necessarily chat rooms, but we make a profile, we talk to them about why we’re here, we’re sex educators, let’s talk about sex, keeping safe, PrEP, all that stuff. Well, I don’t necessarily say it was entirely up to COVID. I mean they were kind of headed in that direction. COVID just pushed it along.” (Test Counselor 7)
2.d. Organizational adaptations to facilities and workspaces	Q9: “We've had to kind of revamp the way we do testing in general…in regards to wearing PPE, maintaining social distancing, pre-screening clients when they come into our agency, with temperature checks and things like that.” (Test Counselor 8)
3. Emerging and ongoing health disparities in the context of COVID-19	
3.a. Increase in HIV disparities attributable to COVID-19	Q10: “Because they [Hispanic population] still need to learn more about PrEP. In my experience of providing testing, especially when it comes to somebody who is from the Hispanic population and background they have no idea that this PrEP is available. If it was like this before the pandemic, now it’s even worse, because they’re not going to be tested.” (Test Counselor 9)

#### Disruptions due to COVID-19

Test counselors reported that COVID-19 caused disruptions in service delivery including: reduction in staff availability and morale, disruptions to organizational networks, challenges transitioning to telehealth, reduction in outreach and available services, and reduction in client access and demand for services.

In terms of *reduction in staff availability and morale*, participants described different types of disruptions related to COVID-19 outbreaks among staff and deaths of colleagues (Table 4: Q1). These challenges, as well as social isolation, layoffs, fears of acquiring COVID-19 at work, insufficient resources for COVID-19 prevention (e.g., personal protective equipment, space for social distancing), lowered staff morale.

Participants explained it was not just their own organizations that faced disruptions, but they also saw *disruptions to their organizational networks* (i.e., other organizations were closed, had limited hours, had to task shift to deliver COVID-19 services), making it harder to reach other organizations, which counselors would normally do to refer clients for PrEP and behavioral health.

*Challenges transitioning to telehealth* were common. Test counselors explained that implementing telehealth was time consuming (e.g., took longer to provide referrals), difficult (e.g., harder to provide test counseling by phone), and made it harder to reach and build rapport with clients, particularly those with limited technology access or unaccustomed to remote interactions (e.g., individuals with lower income, older adults) (Q3). Some described skepticism regarding the effectiveness of telehealth.

Participants reported a *reduction in outreach and available services* during COVID-19. Several reported restrictions on mobile service delivery due to COVID-19 and challenges providing community-based outreach. Counselors noted their resources were limited due to reductions in grants and supply chain issues in obtaining HIV tests due to funding being redirected to COVID-19 from other health services.

Finally, counselors noted *reductions in client access and demand for services*. They explained that some clients seemed a) hesitant to seek HIV services due to COVID-19, b) preferred in-person services and did not prefer telehealth, or c) needed services but were unable to access them during COVID-19 due to structural barriers (e.g., transportation, technology) (Q5).

#### Resilient innovations in the context of COVID-19

Participants also reported that COVID-19 led their organizations to innovate to be able to continue providing needed HIV services in this new healthcare landscape, thereby demonstrating resilience. Innovations in response to COVID-19 included: increased availability of telehealth; increased availability of other remote service options; flexible methods of community-based outreach and engagement; and organizational adaptations to facilities and workspaces for COVID-19 prevention.

Nearly every participant indicated their organization adapted by *offering telehealth services* that were previously unavailable or had limited availability. Despite the challenges of transitioning to telehealth, counselors generally viewed telehealth as a flexible model that would help some clients access behavioral and sexual health services post-COVID-19 (Q6). They also noted telehealth could be easier to implement than in-person services (e.g., omitting the need for a registration process). Additionally, counselors noted *increased availability of other remote service options*, such as mailing HIV testing kits and providing medication delivery, which they saw as valuable beyond COVID-19 (Q7).

Participants described developing *flexible methods of community-based outreach and engagement* during COVID-19. They did this by not only modifying and updating outreach and engagement strategies, but also developing novel strategies to engage clients. Participants explained that their organizations enhanced their mobile outreach and services, increased their app-based outreach, and extended prescriptions to reduce medication gaps (Q8). Finally, participants noted *organizational adaptations to facilities and workspaces for COVID-19 prevention*, such as increasing available PPE and restructuring workspaces, enhancing their comfort working on site (Q9).

#### Health Disparities

The final qualitative theme was *emerging and ongoing HIV disparities attributable to COVID-19*. Through their work, counselors recognized that COVID-19 disproportionately impacted access to HIV related services among key populations, including racial/ethnic minorities, people with lower SES, people experiencing homelessness, and older adults (Q10).

## Discussion

This mixed methods study revealed that although test counselors in MDC faced disruptions in the context of COVID-19, they responded with innovation that might point to potential strategies for building health system resilience beyond the acute COVID-19 pandemic, including new variants, surges, and new emerging infectious diseases. Our findings align with the National HIV/AIDS Strategy, which calls for maintaining COVID-19 related innovations to continue promoting access among key populations [29]. Furthermore, the EHE plan calls for innovative solutions to enhance implementation of HIV prevention and treatment to ensure reach to those most affected by HIV. The findings from the current study reveal potential innovations, including new implementation of alternative service delivery models (e.g., telehealth, mail or person-to-person delivery of medications and tests) that could facilitate scaling out services to those underreached prior to COVID-19. These findings build upon prior studies showing promising innovations in HIV service delivery during COVID-19, including developing telemedicine for HIV prevention and care, providing services by phone, providing curbside services, and increasing provision of ancillary services such as food and personal care products [14, 17, 30].

Despite the promise of learning from the innovations developed during COVID-19, care must be taken in ensuring these innovations do not come at the cost of equity, which is relevant to our qualitative theme of emerging and ongoing health disparities during COVID-19. Studies have shown innovations such as the rapid transition to telemedicine can amplify the “digital divide,” which refers to unequal access to technology delivered care [31]. Particularly, older adults, non-English speakers, individuals with lower socioeconomic resources, and individuals in rural areas experience challenges due to lack of technology access, absence of broadband connectivity, and inexperience with remote visit software [32, 33]. Although our quantitative findings suggested that innovations generally exceeded disruptions, it is important to examine whom disruptions and innovations most affected. Our qualitative findings suggested inequitable impacts of COVID-19 on subgroups most impacted by HIV and underscored that new innovations may not equitably meet the needs of all individuals affected by HIV.

Another contribution of this study is that it solicits the perspectives of implementers – individuals who provide HIV testing services – which complements prior work examining how COVID-19 affected HIV services from a consumer perspective. Prior work found that Latino MSM in MDC reported increased barriers to HIV and behavioral health services during COVID-19 due to factors including lack of knowledge about where to get services due to organization closures, privacy concerns, and financial barriers, whereas remote services enhanced access [15]. Current findings from an implementer perspective largely showed convergence between implementers and consumers, while also extending prior findings by highlighting the unique experiences of implementers and describing how organizations innovated during this period. Our findings highlight the utility of multilevel perspectives in future research.

**Fig. 3:**
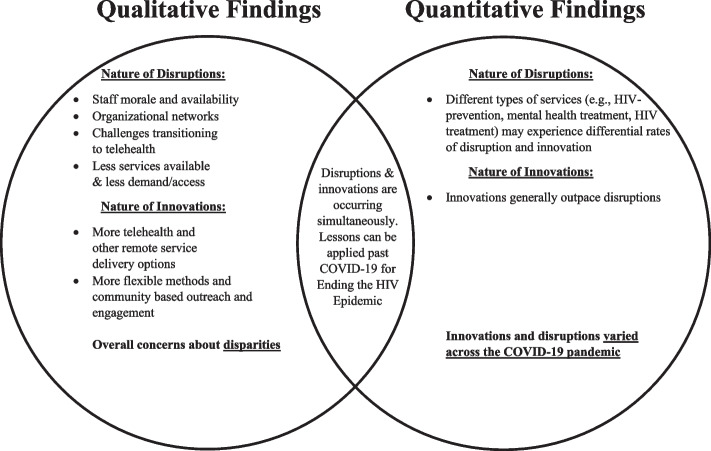
Integration of Qualitative and Quantitative Findings

Our findings also draw on strengths from quantitative and qualitative methods. Figure 3 illustrates areas of convergence and divergence between data sources. While the qualitative data provided an in-depth understanding of the nature and types of disruptions, the quantitative data showed that despite these disruptions, they were generally outpaced by innovative responses. The qualitative data further unpacks these quantitative findings by revealing that innovations might not equitably serve all HIV-affected communities.

Despite the strengths of this mixed method study, it has limitations. We had a lower than expected survey response rate potentially due to the stress counselors experienced during COVID-19, their increased workload, and the change in recruitment described above. Although we examined differences across time with our cross-sectional data, data was collected from during a time in which changes occurred with respect to COVID-19 (e.g., vaccines, changes in shelter in place order, fluctuating COVID-19 incidence). Although we attempted to illustrate the temporal patterns in the sample, time was not a design factor in this survey and multiple factors may have caused these patterns. Additionally, we had very limited time to collect data in the context of a required training for HIV test counselors; therefore, we were unable to collect some counselor-level information such as facility-level affiliation (for organizations with multiple facilities) or other relevant demographics about counselors. We strived to fill these gaps by collecting additional details about counselors in the context of the qualitative interviews which were not as time constrained. Finally, we note that although HIV test counselors were likely aware of other HIV-related service disruptions and innovations given their central client-facing role in organizations, it is possible that a more comprehensive study that included individuals in other roles (e.g., case managers, medical providers) could have provided unique insights into the extent to which HIV services within their specialty role were innovated or disrupted. Although it is possible that HIV test counselors underreported innovations or disruptions for services with which they were less familiar, our prior work showed that Latino sexual minority men reported less disruption of HIV treatment services compared to HIV prevention services [15], which is consistent with our current findings.

Our findings point to potential changes in policy and practice that could be maintained beyond the immediate impacts of COVID-19 to build HIV service organizations’ resilience to future disruptive events. For example, service delivery and outreach innovations remedied patients’ transportation related barriers and wait times. Organizations adapted to COVID-19 by offering telehealth services, which allowed for continued HIV testing, PrEP, and behavioral health services and referrals amidst pandemic related closures. Telehealth offers a flexible model for accessing care after the pandemic, or in future infectious disease pandemics. Federal and local HIV service funders could consider support for smartphones, tablets, and technology support for organizations and patients, which could help to overcome digital divides. Novel outreach and service delivery methods such as mobile or street-based testing and outreach, app-based outreach, and extended prescription policies beyond COVID-19 would allow organizations to reach a larger patient population and address potential lapses in prescriptions. National pharmacy data indicate that after the US declared the pandemic a national emergency, there was a 22% reduction in PrEP prescriptions and 25% decrease in PrEP initiation [34]. Extending prescriptions could prevent medication gaps and minimizes the need to re-engage patients in a PrEP regimen.

Implementation strategies to support the sustainment of the innovations observed in our study may be needed, such as accessing new funding, simplifying billing and/or reducing fees for remote service delivery, providing technical assistance, and using mass media and other outreach strategies to enhance the reach of innovative service delivery models to those who could benefit most [35]. Additionally, implementation research will be needed as the innovations we observed in the current study roll out and are sustained over time to evaluate the extent to which they might mitigate or exacerbate disparities [36]. In the case that innovations exacerbate disparities, which is possible based on our qualitative findings, implementation strategies that seek to achieve equity could support achieving the EHE goals while learning from the COVID-19 pandemic.

In sum, the current study highlights both the disruption to HIV services that occurred due to COVID-19 in MDC, as well as the ways organizations demonstrated resilience through innovation to meet the needs of HIV-affected communities. As observed in our data, focused attention is needed to ensure that innovations yield equitable benefit, as the HIV epidemic has long been driven by disparities. Lessons learned from the COVID-19 pandemic can inform innovative strategies for improving the reach of HIV services to underserved communities and facilitate reaching EHE goals during and beyond the current COVID-19 pandemic.

## Data Availability

Qualitative data is not available as it is potentially identifying. Quantitative data (without identifiers) are available to researchers upon reasonable request (e.g., methodologically sound proposal and signed data use agreement) from the corresponding author, AH aharkness@miami.edu, following publication. Analyses with this data may only be used to achieve the aims in the approved proposal.
